# Lithium Counselling in Women of Childbearing Age

**DOI:** 10.1192/bjo.2022.449

**Published:** 2022-06-20

**Authors:** Sarah Jeyaprakash, Kyla Ng Yin, Isabella Broughton

**Affiliations:** 1 North East London NHS Foundation Trust, London, United Kingdom; 2 Barking, Havering and Redbridge University Hospitals NHS Trust, London, United Kingdom; 3 North East London Foundation NHS Trust, London, United Kingdom; 4 Barts Health NHS Trust, London, United Kingdom

## Abstract

**Aims:**

Lithium is a commonly prescribed mood stabiliser given to women of childbearing age. There are risks of teratogenicity in first trimester of pregnancy, most notably cardiac abnormalities. It is not clear whether this is highlighted to patients. Our aim was to evaluate whether women were being counselled according to NICE and BNF guidelines.

**Methods:**

We analysed records for 25 female inpatients who were commenced on lithium in Goodmayes Hospital from August to September 2021 to see if lithium counselling was done and documented on Rio. This was corroborated with e-prescribing records on ePMA.

**Results:**

Data were collected from 26 patients; 1 was post-menopausal (excluded), final sample size n = 25. 16% were given a lithium leaflet, 92% had trialled alternative antipsychotics, 8% were asked if planning pregnancy, 4% had the risks of lithium in pregnancy explained and 12% were offered contraception.

**Conclusion:**

Lithium counselling needs to improve. We should give patients information via lithium leaflets and explain the risks when they improve in mental state. We should arrange contraception referrals if desired and signpost perinatal psychiatry team if planning a pregnancy.

